# The Responses of Alternative Splicing during Heat Stress in the Pacific White Shrimp *Litopenaeus vannamei*

**DOI:** 10.3390/genes14071473

**Published:** 2023-07-19

**Authors:** Xiaoxi Zhang, Xiaojun Zhang, Jianbo Yuan, Fuhua Li

**Affiliations:** 1 Chinese Academy of Sciences (CAS) and Shandong Province Key Laboratory of Experimental Marine Biology, Institute of Oceanology, Chinese Academy of Sciences, Qingdao 266071, China; zhangxiaoxi@qdio.ac.cn (X.Z.); xjzhang@qdio.ac.cn (X.Z.); yuanjb@qdio.ac.cn (J.Y.); 2 Key Laboratory of Breeding Biotechnology and Sustainable Aquaculture, Chinese Academy of Sciences, Wuhan 430072, China; 3 Center for Ocean Mega-Science, Chinese Academy of Sciences, Qingdao 266071, China

**Keywords:** alternative splicing, heat stress, *Litopenaeus vannamei*, RNA-Seq

## Abstract

Heat tolerance is increasingly becoming a crucial trait for aquaculture species in the face of rapidly changing climate conditions. Alternative splicing (AS) is a vital mechanism within cells that modulates gene abundance and functional diversity, enabling organisms to effectively respond to diverse stressful conditions, including thermal stress. However, it is still uncertain whether AS contributes to heat tolerance in shrimp. In this study, we conducted an extensive transcriptome analysis on the Pacific white shrimp, *Litopenaeus vannamei*, revealing a total of 1267, 987, and 130 differential AS events (DAS) in the gill, hepatopancreas, and muscle, respectively, following exposure to heat stress. Among all of the DAS events, exon skipping (ES) was the predominant form of splicing modification observed. Interestingly, a minor portion of DAS genes exhibited overlap across the three tissues, implying that heat stress exerts unique effects on various tissue types. Moreover, the functional enrichment analysis demonstrated that commonly identified DAS genes were primarily associated with the “spliceosome” pathway, indicating that the AS of splicing-related genes played a crucial role in the response to heat stress. Our findings also revealed that heat stress tended to induce longer mRNA isoforms through differential alternative 3′ splice site (A3SS) events. Notably, A3SS events exhibited the highest proportion of maintained open reading frames (ORFs) under heat stress. Interestingly, we observed a limited overlap between the genes exhibiting DAS and those showing differential gene expression (DEG), indicating that AS may function as a distinct regulatory mechanism independent of transcriptional regulation in response to heat stress. This is the first comprehensive study on AS in crustacea species under heat stress, which broadens our understanding of the regulatory mechanisms governing the crustaceans’ response to environmental stress, providing valuable insights for the aquaculture breeding of shrimp and other aquatic animals.

## 1. Introduction

Alternative splicing (AS) is a prevalent post-transcriptional process found in eukaryotes, which enables the production of multiple mRNA variants from a single precursor mRNA (pre-mRNA) by utilizing distinct splice sites [1]. Splice site selection is known as a crucial factor in determining the splicing outcome. Splice site recognition primarily relies on the assembly of the spliceosome on the pre-mRNA, whereas splice site selection is influenced by regulatory sequences present within the mRNA itself (cis-acting regulatory sequences) as well as external factors (trans-acting factors) [2]. Regulatory splicing factors, including serine/arginine-rich (SR) proteins and heterogeneous nuclear ribonucleoproteins (hnRNPs), recognize and interact with cis-acting elements [3]. The interplay between cis-elements and the expression of splicing factors are responsible for the diversification of AS patterns, resulting in a remarkable increase in organisms’ transcriptome complexity and proteome diversity [4,5]. Based on current knowledge, AS can be classified into at least five distinct types: exon skipping (ES), intron retention (RI), alternative 5′ splice site (A5SS), alternative 3′ splice site (A3SS), and mutually exclusive exon (MXE) [3]. ES refers to the process where a specific exon is excluded from the pre-mRNA during splicing. RI refers to the phenomenon where an intron is not excised from the pre-mRNA and instead remains present in the mature mRNA. A5SS and A3SS involve the spliceosome utilizing alternative 5′ and 3′ splice sites, respectively. MXE occurs when one of two exons is retained while the other is spliced out exactly [6]. Moreover, the amalgamation of these fundamental AS types is widely recognized as the underlying mechanism behind the intricated and complex AS patterns observed in various living organisms [7]. In recent years, the rapid advancements and widespread application of high-throughput sequencing technology have enabled the genome-wide analysis of AS in numerous animal species. Based on existing research findings, it has been observed that around 95% of multi-exon genes in humans [8,9], 40% in of multi-exon genes *Litopenaeus vannamei* [10], 31% of multi-exon genes in the fruit fly (*Drosophila melanogaster*) [11], 25% of multi-exon genes in nematodes (*Caenorhabditis elegans*) [12], and 16% of multi-exon genes in oysters (*Crassostrea gigas*) [13] undergo AS. It has been demonstrated that modulation of isoform expression through AS also plays crucial roles in response to diverse stressful conditions. For instance, *D. melanogaster* exhibits temperature-dependent regulation of isoform expression in the timeless gene, resulting in the generation of a temperature-specific timeless function [14]. Likewise, in the oyster *Crassostrea virginica*, the abundance of two isoforms of the alternative oxidase (AOX) gene, generated through A3SS of the tenth exon, undergoes a switch under hypoxic conditions compared to normal conditions [15]. Other studies have revealed that the acute exposure of *Daphnia pulex* to copper elicits notable and extensive alteration in AS, underscoring the indispensable role of AS in modulating the diversity of the transcriptome in response to metal exposure in Daphnia [16].

The Pacific white shrimp, *L. vannamei*, is one of the most economically significant shrimp species worldwide, with it being extensively produced throughout mariculture. In comparison to other penaeid shrimps, *L. vannamei* exhibits a higher lethal temperature (approximately 40 °C), suggesting that it possesses a more adaptable mechanism to withstand the stress caused by elevated temperatures. Our previous study reported that pathogen infection or biotic stress increased the number of AS events in *L. vannamei*, which suggests that shrimp adopts AS as a strategy to resist environmental stimulus [10]. Furthermore, mounting evidence highlights the significant roles of AS during heat shock in various species. For instance, heat stress has been found to induce alterations in the global AS patterns in fruit flies [17], fish [18], and nematodes [19]. In this context, it is plausible to speculate that shrimp also employ AS modulation as a response mechanism to heat stress.

In this study, we investigated the dynamic changes of AS in *L. vannamei* in response to heat stress through RNA-Seq analysis. The obtained results enabled us to identify and characterize all differential AS events (DASs) under heat stress response and their corresponding genes (DASGs). To gain insights into the biological processes associated with AS, we compared the AS patterns among three tissues (muscle, gill, and hepatopancreas) and performed functional enrichment analysis on DASGs. Moreover, we compared DASGs and differentially expressed genes (DEGs) to elucidate the impact of AS on gene expression. Finally, we examined the impact of AS on gene function by analyzing the frameshift ratio.

## 2. Materials and Methods

### 2.1. Data Source and AS Event Identification

Transcriptomic data of *L. vannamei* under heat stress were obtained from our previous study [20], which were brought from the gill, hepatopancreas and muscle. The data of 25 °C and 33 °C served as the control and heat stress group, respectively. Clean reads were archived by removing low-quality reads and adapter sequences using fastp (v0.20.1) [21]. Then, the trimmed reads of each sample were mapped to the shrimp genome [22] using STAR (v2.7.9a) with default parameters [23]. Finally, the DAS events in each tissue under heat stress were identified by comparing the transcriptomic profiles with the control group using rMATS (v4.1.0) software with default settings [24]. The dynamic changes in the AS profile were estimated by calculating the percentage spliced-in (PSI) values for each AS exon. AS events with a ΔPSI > 0.1 and an adjusted *p*-value < 0.05 were classified as DAS. Conversely, AS events with a ΔPSI < 0.1 or adjusted *p*-value > 0.05 were categorized as unaffected AS events (UAS). The AS events were visualized by rmats2sashimiplot. To assess the potential impact of heat-induced AS on gene function, we identified DAS events that potentially disrupt the open reading frame (ORF) by examining whether the length of skipped exons is divided by three [25].

### 2.2. Transcriptome Assembly and Differential Expression Analysis

Clean reads were mapped to the *L. vannamei* genome by Hisat2 software, and transcripts were assembled using StringTie [26]. To estimate the expression abundance of the transcripts, the FPKM (fragment per kilobase of transcript per million mapped reads) values of the transcripts were calculated using StringTie. The differential expression analysis was performed by DESeq2 [27]. The genes/transcripts with adjusted *p* < 0.05 and |log2(fold change)| > 1 were considered as DEGs. Principle component analysis (PCA) was performed using the online website OmicShare (http://www.omicshare.com/tools, accessed on 7 March 2021). The Venn diagrams were visualized by TBtools [28].

### 2.3. Gene Function Enrichment Analysis

To investigate the associated biological processes or pathways of DAS genes, the functional enrichment analysis was conducted with the following steps. First, Gene Ontology (GO) and Kyoto Encyclopedia of Genes and Genomes (KEGG) annotation of genes were generated by eggNOG-mapper [29]. Then, the functional enrichment analysis of the DAS genes was performed using OmicShare. The calculated *p*-values were corrected by FDR. Finally, the GO terms or pathways with an FDR ≤ 0.05 were defined to be significantly enriched.

## 3. Results

### 3.1. Overview of AS Events

**Figure 1:**
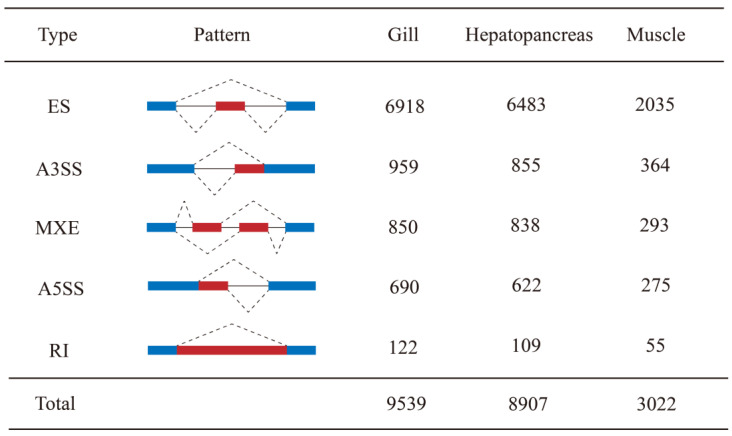
The AS pattern and the corresponding number of AS events detected in three tissues under heat stress. The blue and red boxes represent fixed exons and the alternative spliced exons, respectively. ES: exon skipping, A3SS: alternative 3′ splice site, MXE: mutually exclusive exon, A5SS: alternative 5′ splice site, and RI: retained intron.

A total of 9539, 8907, and 3022 AS events were identified in the gill, hepatopancreas, and muscle under heat stress, respectively (Figure 1). Although the total number of AS events varied greatly, the distribution profile of five types of AS events (ES, A3SS, A5SS, MXE, and RI) was consistent among the three tissues. In detail, ES was the most abundant event, accounting for 72.52%, 72.79%, and 67.3% of total AS events in the gill, hepatopancreas and muscle, respectively, succeeded by A3SS, MXE, and A5SS. The occurrence of RI was observed in only 1.28%, 1.22%, and 1.82% of AS events across the three respective tissues.

### 3.2. Identification of DAS Events and Their Related Pathways under Heat Stress

**Figure 2:**
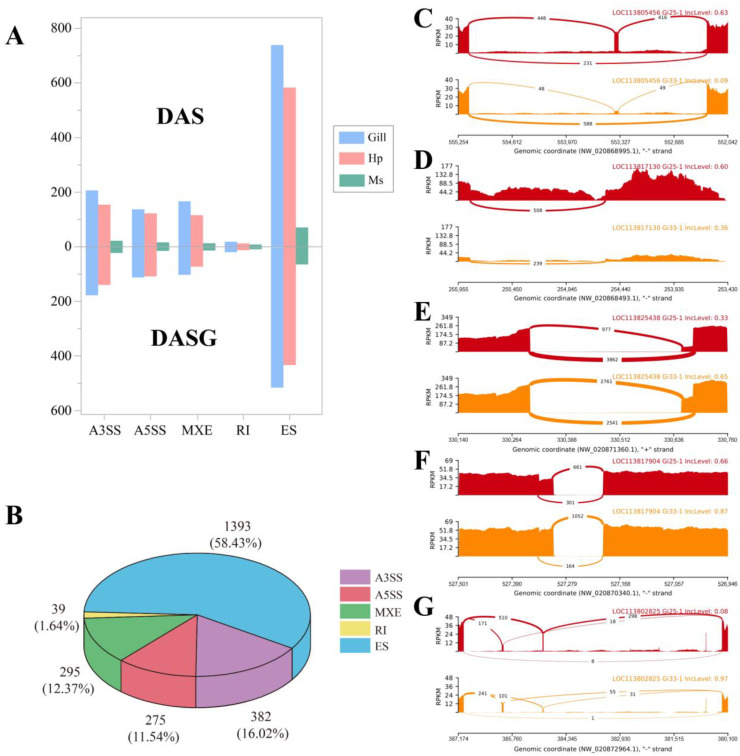
(**A**) The number of DASs (above) and DASGs (below) in three tissues. (**B**) The pie chart of the total number and ratio of DASs for different AS types. (**C**–**G**) The visualization of representative DASs for (**C**) the adenylyl-cyclase-associated protein 1 gene occurring ES, (**D**) sushi, von Willebrand factor type A, EGF, and pentraxin domain-containing protein 1 gene occurring RI, (**E**) eukaryotic translation initiation factor 4 gamma gene occurring A3SS, (**F**) heterogeneous nuclear ribonucleoprotein U-like protein 1 gene occurring A5SS, and (**G**) glucosamine-fructose-6-phosphate aminotransferase 2 gene occurring MXE using rmats2sashimiplot. The red and orange colors represented the control and heat stress groups, respectively.

DAS events and their corresponding genes (DASGs) were identified in all three pairwise comparisons under heat stress conditions. The results showed that a total of 1267, 987, and 130 DAS events were characterized in the gill, hepatopancreas, and muscle with the cut-off of ΔPSI > 0.1 and adjusted *p* < 0.05, respectively (Figure 2A). Generally, they accounted for 13.28%, 11.08%, and 4.3% of the total AS events in the corresponding tissues. DAS events in the three tissues were involved in 769,661 and 107 DASGs, with an average of 1.64, 1.49, and 1.21 AS isoforms per gene. These results showed that the AS of the gill was most sensitive to heat stress, followed by that of the hepatopancreas and muscle. In terms of the different AS types, ES was also the most abundant DAS event representing 58.43% of total DAS events, followed by A3SS (16.02%), A5SS (11.54%), MXE (12.37%), and RI (1.64%) (Figure 2B). The representative DAS events of adenylyl cyclase-associated protein 1 (*CAP1*), sushi, von Willebrand factor type A (*VWA*), EGF and pentraxin domain-containing protein 1 (*SVEP1*), eukaryotic translation initiation factor 4 gamma (*EIF4G1*), heterogeneous nuclear ribonucleoprotein U-like protein 1 (*HNRNPUL1*), and glucosamine-fructose-6-phosphate aminotransferase 2 (*GFPT2*) genes were visualized using rmats2sashimiplot (Figure 2C–G).

**Figure 3:**
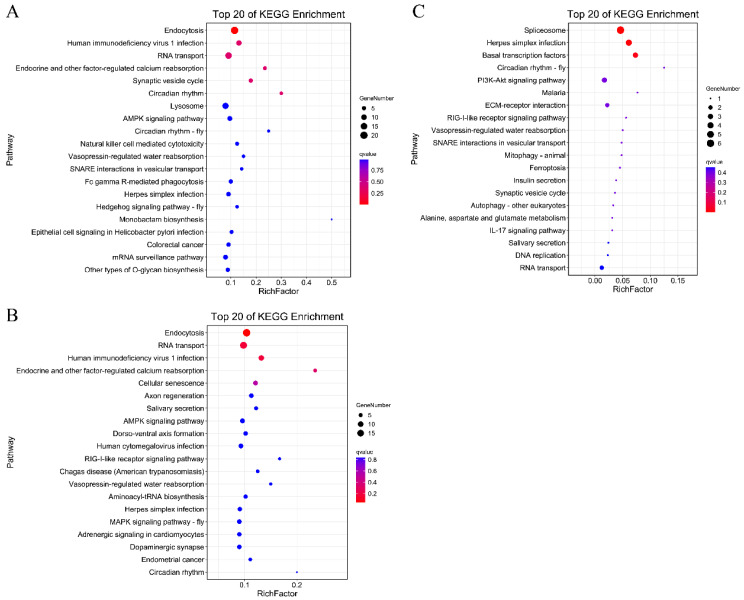
The KEGG enrichment analysis of DASGs in the (**A**) gill, (**B**) hepatopancreas, and (**C**) muscle. The circle size and color represent the number of DASGs and the q-value of the corresponding pathway, respectively.

The KEGG enrichment analyses of the DAS genes were conducted to investigate the role of AS in response to heat stress. The result revealed that the DASGs of the gill and hepatopancreas were both significantly enriched at “endocytosis” (ko04144) and the “RNA transport” (ko03013) pathway (Figure 3A,B). As for the muscle, the DASGs were signicantly enriched at “spliceosome” (ko03040) and the “basal transcription factors” (ko03022) pathway (Figure 3C).

### 3.3. Different Tissues Possessed Distinct AS Regulation Patterns under Heat Stress

**Figure 4:**
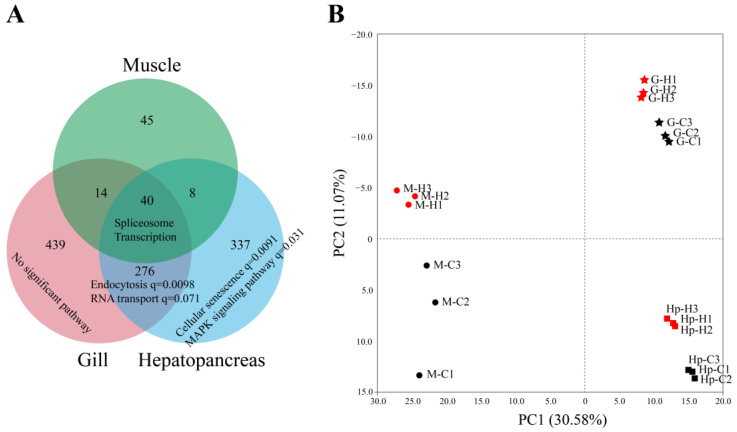
The relationship between the AS patterns among the three tissues. (**A**) The Venn diagram for the DASGs of the three tissues under heat stress. The enriched or main pathways of all gene sets were visualized below the number of DASGs. (**B**) The principal component analyses based on the PSI values of all common AS events among three tissues. The black and red points represent control and heat stress groups, respectively. The circles, squares, and stars represent the muscle, hepatopancreas, and gill, respectively.

To figure out whether heat stress had different effects on the AS profile of different tissues, we compared their DAS events and DAS genes. The results showed that three tissues only shared 40 DASGs (Figure 4A). Notably, more than one-third of them were related to transcription and pre-mRNA splicing, like RNA-binding proteins, splicing factors, and transcription factors (Figure 3A, Table 1). Moreover, the gill and hepatopancreas contained a large number of specific DASGs. The DASGs shared by the gill and hepatopancreas were significantly enriched in “endocytosis” and the “RNA transport” pathway, and the specific DASGs of the hepatopancreas were mainly related to “cellular senescence” and the “MAPK signaling pathway”. These results indicated that AS regulation in response to heat stress was different among the three tissues, which was also supported by the PCA analysis of the PSI values of all common AS events. The PCA plot revealed an apparent separation for different tissues. In detail, PC1 separated the muscle and other tissues, and PC2 separated the gill and hepatopancreas.

### 3.4. Heat Stress Prefered Longer Isoforms Occurring A3SS

**Figure 5:**
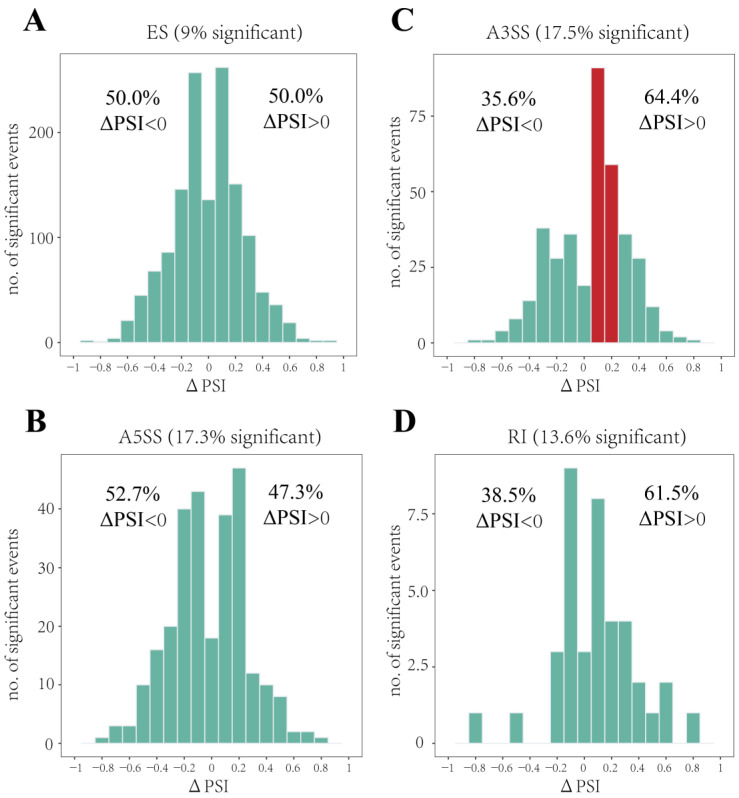
The distribution pattern of the PSI values of each type of AS under heat stress. (**A**) ES, (**B**) A5SS, (**C**) A3SS, and (**D**) RI. The *y*-axis represents the number of DAS events. The ratio of DAS events is shown in each figure. The red bars in (B) represent the obvious biases toward the positive values.

The distribution pattern of the PSI values for ES, A3SS, A5SS, and RI was analyzed to estimate the effect of heat stress on the length of transcribed mRNA. A ΔPSI greater than 0 indicates an increased proportion of the long isoform (inclusion) of a gene, while the short isoform (exclusion) decreased in response to heat stress. The results showed that the inclusion and exclusion isoforms shared a similar number and almost symmetrical distribution for ES and A5SS (Figure 5A,B), which suggested that heat stress barely affected the length of transcripts occurring ES and A5SS. Notably, A3SS showed substantially biases toward the positive values after heat stress (64.4% of ΔPSI > 0, Figure 5C). In particular, there were 150 DAS events with a ΔPSI value ranging from 0.05–0.25 (Figure 5C, red bars), which accounted for 39.16% of total DAS events occurring A3SS. Whereas, the distribution pattern of RI was meaningless owing to its small number (Figure 5D).

### 3.5. Regulation of AS and Transcription in Response to Heat Stress

**Figure 6:**
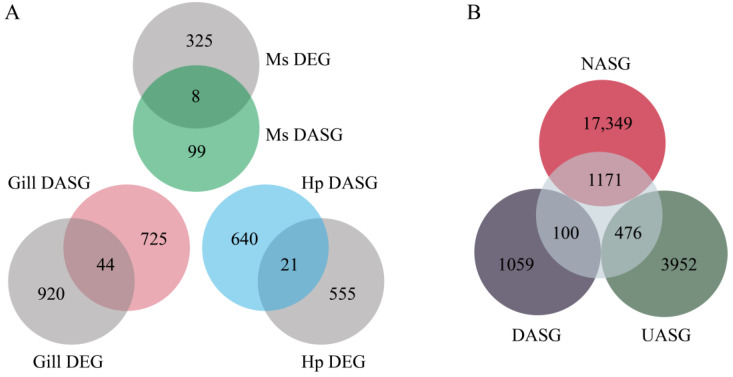
The relationship between AS and gene expression. (**A**) Venn diagram of the overlap of the DEGs and DASGs in the three tissues. (**B**) Venn diagram of the overlap of the DEGs, DASGs, UASGs (unaffected AS genes), and NASGs (non-AS genes) under heat stress. The inner circles represent the DEG gene sets.

Generally, the PCA analysis showed that the control groups were both clearly separated from the heat stressed groups based on the FPKM or PSI values (Figure 4B), suggesting that gene expression and AS both contribute to heat resistance. To further determine the relationship between AS and gene transcription, we compared DEGs with DASGs in each tissue. A total of 964, 576 and 333 DEGs were characterized in the gill, hepatopancreas and muscle under heat stress, respectively. However, only 44, 21, and 8 DEGs overlapped with DASGs in the three tissues(Figure 6A), suggesting that these co-regulated genes account for a small portion of DEGs or DASGs in shrimp. In addition, the DEGs were compared to non-AS genes (NASGs), unaffected AS genes (UASGs), and DASGs to further determine the influence of AS on gene expression. The results showed that DEGs shared the greatest overlap with NASGs, accounting for 67.03% of total DEGs, followed by UASGs (27.25%). However, only 5.72% of the total DEGs overlapped with the DASGs (Figure 6B). These results suggest that AS had a limited effect on gene expression under heat stress, and they could be separately regulated in response to heat stress in shrimp.

### 3.6. The Effect of AS on Gene Function under Heat Shock

To explore the potential impacts of heat-induced AS on gene function, the frameshift of all transcripts was analyzed. Generally, except for RI, a high proportion of maintained ORF for all AS types ranged from 55.28–86.65% (Table 2). Among them, A3SS possessed the maximum ratio of maintained ORFs, up to approximately 87%. However, only 20.5% of the DAS transcripts maintained their original ORFs when RI occurred. The KEGG enrich analysis results showed that those DAS genes that maintain ORFs were significantly enriched in the “endocytosis” pathway, which was similar to the KEGG enrichment analysis of total DAS genes. Meanwhile, DAS genes occurring frameshift were related to the “aminoacyl-tRNA biosynthesis” pathway (ko00970).

## 4. Discussion

Since shrimp are subjected to various environmental stresses during their growth and development, they have evolved a robust adaptive mechanism at both transcriptional and post-transcriptional levels. However, the current understanding of transcriptomic responses to stress primarily revolves around the transcriptional level, leaving the post-transcriptional regulation aspect largely unexplored and poorly understood. In fact, the dynamic changes in the AS profile have been extensively elucidated under various stress conditions in shrimp and other crustacean species [10,16,30,31], providing valuable insights into the significant role of AS in the adaptive evolution of animals. However, the extent to which AS contributes to heat stress resistance in shrimp remains uncertain. In this study, we utilized RNA-Seq data to investigate the role of AS regulation in response to heat stress in *L. vannamei*.

### 4.1. The Effect of Heat Stress on the AS Profile of Shrimp

Under heat stress, ES is the most abundant DAS event, followed by A3SS, MXE, A5SS and RI. This is consistent with the global AS pattern of *L. vannamei* [10] and other animals [8,11,13], implying conservation of AS profile in animals. As the rarest AS event, RI also has the lowest ratio of maintained ORFs. Except for RI, other AS types retained a high proportion of DASG with maintained ORFs under heat stress in the shrimp. It seems that the DAS of shrimp contributes to the structural changes, rather than disrupting the gene function by frameshifts under heat stress. Consistently, a high proportion of DAS events with maintained ORFs (62.45–75.81%) was found in the sea squirt *Ciona savignyi* under heat stress, which tolerates acute environmental changes during invasion [32].

Generally, the average length of AS genes is significantly larger than that of non-AS genes because of a greater number of exons and a longer intron length [10]. In addition, the transcript length can be influenced by high temperature through DAS. For example, mouse fibroblasts prefer the longer transcripts occurring RI under heat shock [33], and ES is substantially skewed toward exon loss *C. savignyi* under heat stress [32]. Consistently, the present study revealed an increase in the length of transcripts associated with differential A3SS events in response to heat stress. However, the differential A3SS did not trigger the nonsense-mediated RNA decay (NMD) pathway, as it maintained a high proportion of ORF. Consequently, these events are likely to contribute to functional change through structural or domain gain, thus enhancing phenotypic plasticity in response to environmental stress.

### 4.2. The Self-Alternative Splice of Splicing Factors and RNA-Binding Proteins Plays a Crucial Role in Conferring Heat Resistance

RNA splicing is always accompanied by the RNA binding process, which necessitates the involvement of numerous RNA-binding proteins (RBPs) [34]. Splicing decisions depend on the assembled spliceosomes, the interaction and combination of splicing factors and RNA-binding proteins (SF-RBPs) [35]. In the present study, half of the DASGs shared by three tissues were SF-RBP genes. In addition, 11 of the 54 splicing factors and 10 of the 58 RBPs themselves were differentially alternatively spliced, but few SF-RBPs were differentially expressed under heat stress. These results indicate that differential AS, rather than differential expression, of SF-RBPs plays an essential role in response to heat stress for penaeid shrimp. Similarly, the DASGs were significantly enriched at ‘splicing’ or ‘spliceosome’ processes in response to heat stress in several fish [18,36,37]. This finding suggests the common involvement of auto-splicing or cross-splicing mechanisms in splicing-related genes, highlighting their role in conferring resistance to high temperatures. These findings were also consistent with the observations that splicing factors themselves often undergo auto- or cross-regulation by AS in response to environmental stress in vertebrae [38]. In particular, three serine/arginine-rich genes (SR) (SRSF2, SRSF3, and SRSF7) and a hnRNP protein gene underwent DAS in three tissues under heat stress. It is well known that SR and hnRNP proteins serve as the main splicing factors involved in constitutive as well as regulated alternative splicing [39], and a majority of SR genes exhibited DAS in response to stress [40]. For instance, heat stress induced the DAS of SR and hnRNP genes, which may in turn regulate the AS of other pre-mRNAs and lead to a difference in candidate gene expression between heat-sensitive and -tolerant catfish [18].

### 4.3. Separate Regulatory Mechanisms of AS and Transcription in Response to Heat Stress

Recent studies suggest that both alterations in AS and gene expression potentially contribute to phenotypic variation [41]. Although AS and transcription occur simultaneously within the cell nucleus (co-transcriptional splicing) and splicing efficiency depends on mRNA transcription [42], empirical evidence from a few studies suggests that these two mechanisms are proposed to be separate and parallel processes functioning independently [25,43]. For example, little to no overlap between DASGs and DEGs has been reported in response to heat stress in *D. melanogaster* [17]. The results of our research, demonstrating that only a small subset of DASGs undergo transcriptional modulation in penaeid shrimp, are consistent with previous observations highlighting the independent regulation of these two mechanisms. Moreover, the DASGs and DEGs were mainly associated with distinct biological processes or pathways. Specifically, the DASGs exhibited significant enrichment in “endocytosis” and “RNA transport”, while the DEGs were primarily associated with “glutathione metabolism” in the gill [20]. In our study, heat shock proteins were notably upregulated and enriched, yet the majority of them did not undergo differential alternative splicing. Consistently, the AS pattern of stress-responsive DEGs involved in oxidation reduction and protein folding was found to be unaffected by heat shock in mammal cells [33]. In contrast, several studies have demonstrated that pre-mRNA splicing was co-transcriptionally regulated and that the AS of genes can be affected by their transcriptional activity in zebrafish, killifish, and stickleback in response to cold conditions [44]. In the present study, as well as in other species, it was observed that the number of DASGs increased in conjunction with the number of DEGs [45,46]. This finding suggests the occurrence of co-transcriptional splicing. Hence, more evidence is needed to clarify the regulatory interplay between AS and gene expression, especially across different tissues, stages, or organisms, which would also be important in understanding the separation or collaboration of two mechanisms at long and short timescales in adaptive and plastic responses.

## 5. Conclusions

In this study, we have disclosed the features of AS in *L. vannamei* under heat stress through a comprehensive transcriptome analysis. ES was the most abundant event of all identified DASs under heat stress. Heat stress had different effects on the AS pattern of three tissues: the gill, hepatopancreas, and muscle, with the fact that a few DASGs were shared among them. Moreover, the DASGs of the gill and hepatopancreas both correlated with endocytosis and RNA transport, but those of the muscle were mainly related to the spliceosome and basal transcription factors. Additionally, we demonstrated that only a few genes were shared between the DASGs and the DEGs, suggesting that AS represents an independent gene regulation mechanism in response to heat stress. Furthermore, the transcript exhibiting differential A3SS was found to be elongated and showed the highest proportion of preserved ORFs. Our research not only expands the alternative splicing database of shrimp but also offers novel insights into the potential roles of AS in response to heat stress.

## Figures and Tables

**Table 1:** The function of common DASGs shared by three tissues related to transcription and pre-mRNA splicing under heat stress.

Gene ID	Description	Function
LOC113823825	Nuclear transcription factor Y subunit alpha-like	Transcription; post-transcriptional regulation; pre-mRNA splicing
LOC113823374	Nuclear transcription factor Y subunit gamma-like	Transcription; post-transcriptional regulation; pre-mRNA splicing
LOC113819830	RNA binding protein fox-1 homolog 1-like	Pre-mRNA splicing
LOC113825931	RNA-binding protein 25-like	Pre-mRNA splicing
LOC113803561	RNA-binding protein squid-like	Pre-mRNA splicing
LOC113803611	SAFB-like transcription modulator	Transcription; pre-mRNA splicing; apoptosis
LOC113820949	Serine/arginine-rich splicing factor 2	Pre-mRNA splicing
LOC113816496	Serine/arginine-rich splicing factor 3-like	Pre-mRNA splicing
LOC113802307	Serine/arginine-rich splicing factor 7-like	Pre-mRNA splicing
LOC113820651	Alternative splicing factor ASF/SF2	Pre-mRNA splicing
LOC113811523	HnRNP protein	Transcription; Post-transcriptional modification
LOC113813300	Transcription initiation factor TFIID subunit 1	Transcription
LOC113827782	Transcriptional repressor p66-beta	Transcription
LOC113808730	Upstream activation factor subunit spp27-like	Transcription

**Table 2:** The number and proportion of DAS events with maintained open reading frames (ORFs) under heat stress.

AS Type	DAS Number	ORF Maintained	% ORF Maintained
A3SS	382	331	86.65%
A5SS	275	191	69.45%
MXE	295	222	75.25%
RI	39	8	20.51%
ES	1393	770	55.28%

## Data Availability

The data presented in this study are openly available on the NCBI Sequence Read Archive (SRA) website at [10.1016/j.ecoenv.2022.113600] (accessed on 7 January 2022), accession number [PRJNA798779].
